# Correction: Roll et al. Conceptualization and Validation of the Occupation Insecurity Scale (OCIS): Measuring Employees’ Occupation Insecurity Due to Automation. *Int. J. Environ. Res. Public Health* 2023, *20*, 2589

**DOI:** 10.3390/ijerph21010005

**Published:** 2023-12-20

**Authors:** Lara C. Roll, Hans De Witte, Hai-Jiang Wang

**Affiliations:** 1 Research Group Work, Organisational & Personnel Psychology (WOPP–O2L), KU Leuven, 3000 Leuven, Belgium; hans.dewitte@kuleuven.be; 2 Optentia Research Unit, Vaal Triangle Campus, North-West University, Vanderbijlpark 1900, South Africa; 3 School of Management, Huazhong University of Science and Technology, Wuhan 430074, China; hjiangwang@gmail.com

## Text Correction

There are some errors in the original publication [1]. “All the citations of the Supplementary Materials in the original text should be changed to Appendix C.” A more detailed explanation is shown below:

There was an error in the original publication under Section 2.3.2 [1]. “Following this quantitative pilot, the items for OCIS were reassessed among the researchers one more time and amended into the final scale (see Supplementary Materials for the final scale).”

A correction has been made to “*2.3.2. Results*”, *paragraph number 4.*

“Following this quantitative pilot, the items for OCIS were reassessed among the researchers one more time and amended into the final scale (see Appendix C for the final scale).”

There were two errors in the original publication under Section 3.1.2 [1]. “Occupation insecurity was measured with the newly developed OCIS scale (see Supplementary Materials). The final scale consists of six items for global and five items for content occupation insecurity. A sample item for global occupation insecurity is “I am worried that my occupation will not be needed anymore in the future due to the advancement of technology”. Content occupation insecurity is measured with, for example, the item: “I expect that my occupation will undergo significant changes due to technological developments”. To ensure that participants were aware of the difference between an “occupation” and a “job”, we added instructions with double-check questions to the participants before presenting the items (detailed instructions available in Supplementary Materials).

Two corrections have been made to “*3.1.2. Measures*”, *paragraph number 2.*

Occupation insecurity was measured with the newly developed OCIS scale (see Appendix C). The final scale consists of six items for global and five items for content occupation insecurity. A sample item for global occupation insecurity is “I am worried that my occupation will not be needed anymore in the future due to the advancement of technology”. Content occupation insecurity is measured with, for example, the item: “I expect that my occupation will undergo significant changes due to technological developments”. To ensure that participants were aware of the difference between an “occupation” and a “job”, we added instructions with double-check questions to the participants before presenting the items (detailed instructions available in Appendix C).

There was an error in the original publication under Section 3.1.4 [1]. “When inspecting the modification indices, it became apparent that allowing the error terms of the two items related to training (C4 and C5 in the final scale, see Supplementary Materials) to correlate would improve model fit.”

A correction has been made to “*3.1.4. Results*”, *paragraph number 2*.

When inspecting the modification indices, it became apparent that allowing the error terms of the two items related to training (C4 and C5 in the final scale, see Appendix C) to correlate would improve model fit.

There was an error in the original publication [1]. “Lack Appendix C in the Appendix section”.

A correction has been made to “*Appendix*”:

## Appendix C. Occupation Insecurity Scale (OCIS)

**Occupation insecurity**—The perceived fear that due to automation one may not be able to remain in paid employment in one’s current occupation, or that the occupation will significantly change.

*Please note*: The two dimensions of occupation insecurity (global and content) are meant to be measured separately. It is not intended to form a composite score.

**To ensure that participants are aware of the difference between an ‘occupation’ and a ‘job’, we recommend using the following instructions:**

The following questions ask you about the future of your occupation in light of technological advancements, by which we mean automation, smart technology, artificial intelligence, and robotics.

An ‘occupation’ is a trade or profession that has been learned in training or through experience. An occupation, therefore, requires a certain professional knowledge. When we talk about the disappearance of an occupation in this questionnaire, we mean that the ‘business line’ disappears. An example of this is a food server at a restaurant. If this food server loses their job, they can still work as a food server at another restaurant. However, if restaurants become automated and the occupation disappears, they will never be able to work as a food server again in the future, and they will have to learn another occupation.

Keep this in mind as you complete the following questions. Read the questions carefully and take your time to answer them. Please do not skip questions and indicate one answer option at a time.

Question: After reading the text above, what is an occupation?

1. Being a food server at the restaurant “Bella Italia”.

If chosen: False, being a food server at the restaurant “Bella Italia” is a specific job. The occupation is “food server”, which has been learned and trained through experience. If “Bella Italia” closes, the food server can find a new job at a different restaurant because their occupation is not limited to any specific workplace.

2. Being a food server by profession, after having been trained and/or gained work experience.

If chosen: That is correct.

**Answer options:**

Strongly disagree (1)

Disagree (2)

Partially agree, partially disagree (3)

Agree (4)

Strongly agree (5)

**Global Occupation Insecurity Scale:** The fear of the whole occupation disappearing.

G1. I am worried that my occupation will not be needed anymore in the future due to the advancement of technology.

G2. I am worried that my occupation might disappear due to automation.

G3. There is a risk that I will have to change my present occupation due to automation.

G4. I think that my occupation will not exist anymore in the future.

G5. I am afraid that I will need to switch to another occupation in the short term (1 to 2 years) due to technological developments.

G6. I am afraid that I will need to switch to another occupation later on in my career (5 to 10 years) due to technological developments.

**Content Occupation Insecurity Scale:** The fear of the occupation becoming significantly different, even if the occupation as a whole may not disappear.

C1. I expect that my occupation will undergo significant changes due to technological developments.

C2. Certain tasks of my occupation will no longer be relevant in the future.

C3. I am certain that my occupational responsibilities will change significantly due to technology before my retirement.

C4. I will need to perform tasks in my occupation in the future, for which I am not well trained at the moment.

C5. I need additional training in technology in order to be able to continue working in my occupation.

The authors state that the scientific conclusions are unaffected. This correction was approved by the Academic Editor. The original publication has also been updated.
